# Spectral weighting for sentence recognition in steady-state and amplitude-modulated noise

**DOI:** 10.1121/10.0017934

**Published:** 2023-05-01

**Authors:** Yi Shen, Lauren Langley

**Affiliations:** Department of Speech and Hearing Sciences, University of Washington, 1417 Northeast 42nd Street, Seattle, Washington 98105-6246, USA shenyi@uw.edu, langleyl@uw.edu

## Abstract

Spectral weights in octave-frequency bands from 0.25 to 4 kHz were estimated for speech-in-noise recognition using two sentence materials (i.e., the IEEE and AzBio sentences). The masking noise was either unmodulated or sinusoidally amplitude-modulated at 8 Hz. The estimated spectral weights did not vary significantly across two test sessions and were similar for the two sentence materials. Amplitude-modulating the masker increased the weight at 2 kHz and decreased the weight at 0.25 kHz, which may support an upward shift in spectral weights for temporally fluctuating maskers.

## 1. Introduction

When recognizing speech in background noise, human listeners can take advantage of the temporal fluctuations in the noise envelope to improve speech understanding. In these situations, listeners may utilize the acoustic inputs into the auditory system differently compared to when the speech is presented in steady-state noise. The current study aims to investigate whether listeners weigh acoustic information across the spectrum differently when the background noise has either a steady-state or fluctuating envelope.

The contributions of various spectral regions to speech understanding are not equal. How listeners weight speech information across frequencies, or the band importance function (the BIF), is the key element in many established models of speech intelligibility, such as the Speech Intelligibility Index (SII) (ANSI, 1997). The SII represents the proportion of speech cues available to the listener, and it is based on the weighted sum of a speech-audibility metric over frequency according to the BIF. If a frequency region is important for speech understanding, then it would be associated with a high weight in the BIF. The BIF reflects how speech cues are distributed across the spectrum (French and Steinberg, 1947; Studebaker and Sherbecoe, 1991). For English, a frequency region near 2 kHz has the greatest concentration of acoustic features that carry articulatory information; therefore, the BIF typically exhibits a prominent peak near 2 kHz. Above or below this frequency, the spectral weight decreases.

The BIF in the SII model is not invariant; it varies with features of the target speech, such as the type of speech material (DePaolis, 1996; Du *et al.*, 2019), the availability of visual information (Bernstein and Grant, 2007; Bernstein *et al.*, 2020), and presentation level (Calandruccio *et al.*, 2016; Shen *et al.*, 2020). Recent research also suggests that the BIF may vary from individual to individual (Shen and Kern, 2018; Varnet *et al.*, 2019; Yoho and Bosen, 2019; Shen *et al.*, 2020). For speech recognition in masking sounds, the BIF also depends on whether the masker is broadband noise or competing speech (Buss and Bosen, 2021). Therefore, it is likely that the BIF may also change when other masker properties are varied. The focus of the current study is the changes in the BIF caused by amplitude-modulating the masker.

For normal-hearing listeners, speech understanding may be substantially improved when temporal fluctuation is introduced to the masking noise (e.g., Miller and Licklider, 1950; Festen and Plomp, 1990; Arlinger and Gustafsson, 1991; Eisenberg *et al.*, 1995; Peters *et al.*, 1998; Jin and Nelson, 2006; Shen *et al.*, 2015). It has been hypothesized that this fluctuating masker benefit (FMB) may be partially explained by the listeners' ability to glimpse speech during the temporal dips in the masker where the short-term target-to-masker ratio (TMR) is higher than the long-term nominal TMR. Models that are based on the evaluation of the short-term TMRs are able to capture the FMB in many test conditions (e.g., Cooke, 2006; Rhebergen *et al.*, 2006; van Schoonhoven *et al.*, 2019; Steinmetzger *et al.*, 2019); however, additional auditory processes, such as stream segregation (e.g., Hopkins and Moore, 2009; Steinmetzger and Rosen, 2015) and modulation masking (e.g., Stone *et al.*, 2012; Fogerty *et al.*, 2016), as well as cognitive factors (e.g., George *et al.*, 2007; Zekveld *et al.*, 2013; Schoof and Rosen, 2014; Nuesse *et al.*, 2018) may also influence the amount of FMB.

The relative importance of high and low frequencies for FMB was initially studied by Oxenham and Simonson (2009). These authors presented sentence-in-noise stimuli through either a low-pass or a high-pass filter. The low- and high-pass filters had the same cutoff frequency, and it was selected so that the same speech recognition performance in steady-state noise would be expected from the two filtering conditions. When the masker was amplitude-modulated using a speech envelope, the achieved FMB was similar for the low- and high-pass filtered stimuli. Therefore, there was no evidence from this study to suggest greater importance of either higher or lower frequency regions for speech understanding in fluctuating maskers. However, when spectral weighting is examined in a finer resolution, it is possible that changes in the shape of the BIF could be observed when the maskers have temporally fluctuating envelopes.

Masker amplitude modulation may be expected to influence the BIF for several reasons.

First, if a certain spectral region plays a key role in glimpsing the target speech in the temporal dips of the masker, then this region may carry a higher weight. Previous studies have demonstrated the importance of the temporal fine structure (TFS) or other pitch-relevant cues to FMB. The TFS information may be important to facilitate the perceptual segregation of the target and masker (e.g., Micheyl and Oxenham, 2010). Removing the TFS from the stimuli using noise- or tone-vocoding reduces the amount of FMB (e.g., Qin and Oxenham, 2003; Hopkins and Moore, 2009). Hopkins and Moore (2010) found that removing the TFS information in various frequency regions had a similar effect on FMB. Madsen *et al.* (2021) studied speech recognition in a single-talker masker under low- and high-pass filtering conditions. When systematically increasing the fundamental-frequency difference between the target and masker talkers from 0 to 4 semitones, similar improvements in speech recognition performance were found for the two filtering conditions. The use of TFS information across a wide range of spectral regions may cause the listeners to assign more even weights across frequencies during speech recognition in fluctuating maskers compared to steady-state maskers.

Second, phonemes occupying different spectral regions may be unequally affected by masker amplitude modulation. For example, vowels typically occupy lower frequency regions compared to consonants. Because of their longer durations (e.g., Fogerty and Kewley-Port, 2009), vowel sounds usually provide the listener with multiple glimpses in rapidly fluctuating maskers. On the other hand, consonants are shorter and may be completely masked by a single envelope peak in the masker. In addition to masking in the audio frequency domain, speech signals in high-frequency regions also contain transients and other rapidly varying acoustic features. These features may be subjected to greater modulation masking or interference from a rapidly fluctuating masker, compared to relatively steady speech features in low-frequency regions (e.g., Füllgrabe *et al.*, 2006). Therefore, under rapid masker fluctuations, the audibility of high-frequency speech sounds may be differentially degraded. As a consequence, for a speech recognition task with a low-context test material (e.g., nonsense syllables), the listener may shift spectral weights toward higher frequencies, because the risk of missing transient, high-frequency phonemic cues would be higher than missing the long-duration, low-frequency cues. On the other hand, for a speech recognition task with a high-context test material (e.g., meaningful sentences), the listener may shift spectral weights toward the more audible low-frequency regions.

## 2. Methods

### 2.1 Subjects

Ten adult listeners (nine females and one male) ranging in age from 18 to 35 years, who were native speakers of American English, were recruited for the current study. Although the number of female listeners was much larger than males in the current cohort of listeners, the BIF estimates obtained in the current study agreed with those collected in a previous study using comparable stimuli and procedure and a more balance group of listeners (Shen *et al.*, 2020; 13 females and 17 males). All listeners had audiometric thresholds at 15 dB hearing level (HL) or lower between 250 and 8000 Hz in both ears, with no significant asymmetry (significant asymmetry defined as 15 dB or more at any one frequency, or 10 dB or more at two adjacent frequencies). All listeners provided informed consent before the data collection began. The experimental protocol was approved by the institutional review board at the University of Washington.

### 2.2 Stimuli

During the experiment, listeners were presented with target sentences and instructed to verbally repeat them. Two sentence materials were used, including the IEEE sentences (Galvin and Fu, 2003) and the AzBio sentences (Spahr *et al.*, 2012). The IEEE sentences consisted of 720 sentences, produced by a male or a female talker. During each trial, the sex of the talker was determined randomly, and the test sentence was drawn from the 720 sentences randomly without replacement. The listener's speech recognition performance was scored based on five keywords in each sentence. The AzBio corpus contained 15 sentence lists. Each sentence list consisted of 20 sentences of various lengths (between 3 and 11 words per sentence) and spoken by various male and female talkers. The listener's speech recognition performance was scored based on all words in each sentence. The target sentences were presented at 65 dB sound pressure level (SPL) from a loudspeaker 1 m away in front of the listener (at 0° azimuth).

On each trial, the target sentence was presented in a simultaneous masking noise. The masker was a 20-talker babble, filtered (using fast-Fourier-transform-based filters) to match the long-term spectrum of speech. When the IEEE sentences were used as the target, two separate masking noises were separately generated for the male and female talkers (i.e., to match the long-term spectra of all 720 IEEE sentences produced by the two talkers). When the AzBio sentences were used as the target, a masker was generated to match to the average spectrum of all sentences in the corpus. The masker was gated on 0.5 s before the onset of the target sentence and gated off 0.5 s after the offset of the target sentence. A 60-s sample of the babble noise was first generated and stored as an audio file. On each trial, a section of the stored audio file was randomly drawn, so that the masker varied from trial to trial.

For each of the IEEE and AzBio materials, the masking noise was either steady-state or amplitude-modulated and was presented from the same loudspeaker as the target. When the masker was amplitude-modulated, a sinusoidal modulator was used with a modulation rate of 8 Hz, a modulation depth of 100%, and an initial phase randomly determined for each trial from a uniform distribution spanning 0 and 2*π*. The 8-Hz modulation rate was chosen because it typically is associated with a high amount of FMB for sentence recognition based on previous studies (Miller and Licklider, 1950; Jin and Nelson, 2006; Shen *et al.*, 2015). It is worth pointing out that although the term “unmodulated masker” was used here to label the condition in which the 8-Hz amplitude modulation was not applied, it does not mean that the unmodulated masker, i.e., the 20-talker babble, did not contain inherent amplitude fluctuations (Stone *et al.*, 2012).

All stimuli were presented at a sampling rate of 44 100 Hz. They were presented to the listeners via a 24-bit sound card (AudioExpress, MOTU, Cambridge, MA), a 24-bit equalizer (UltraCurve Pro DEQ2496, Behringer, Willich, Germany), a power amplifier (XLS 1000, Crown, New Bremen, OH), and a full-range loudspeaker (Mod1, ORB Audio, New York). During the experiment, the listeners were seated in a sound-attenuating booth. The loudspeaker was calibrated using the built-in calibration function of the Behringer UltraCurve Pro equalizer and a measurement microphone (ECM 8000, Behringer). Following calibration, a flat frequency response was achieved for the loudspeaker for frequencies above 100 Hz. During the experiment, the experimenter monitored the listeners' verbal responses from the outside of the booth via a monitoring loudspeaker (M1 Active 520 studio monitor, Alesis, Cumberland, RI) connected to a microphone (ECM 8000, Behringer) located inside the booth.

### 2.3 Procedure

The quick-band-importance-function (qBIF) procedure, as described by Shen *et al.* (2020), was used in the current study to estimate the BIFs under various stimulus conditions, with a few modifications. During the qBIF procedure, the listener is presented with sentence stimuli with a simultaneous masking noise at a certain long-term TMR on each trial. The combined speech-in-noise stimulus is presented through a subset of filters in an octave-frequency filter bank (center frequencies: 0.25, 0.5, 1, 2, and 4 kHz). The qBIF procedure improves the efficiency in obtaining estimates of the BIF by iteratively optimizing the choice of the long-term TMR and the bands to present the stimulus on a trial-by-trial basis.

Each run of the qBIF procedure consists of an initial accommodation phase and an active-learning phase, and the selection of the stimulus parameters differs in the two phases. In the accommodation phase, the stimulus is presented through four of the five bands, selected at random. The long-term TMR is decreased by 10 dB when more than 50% of the keywords in the test sentence are corrected recognized for the last two consecutive trials, and it is increased by 5 dB when less than 50% of the keywords are recognized in the last trial. The accommodation phase begins with a long-term TMR of 15 dB and ends when the adaptive track for long-term TMR reaches 16 reversals (typically ∼35 trials). During the active-learning phase of the qBIF procedure, following the termination of the accommodation phase, the stimulus parameters (i.e., the long-term TMR and the bands to present the stimulus) are optimized to maximize the expected information gain or entropy loss. The evaluation of the entropy is based on the posterior parameter distribution of the SII model, refitted to all data collected earlier in the qBIF procedure following each trial [see Shen and Kern (2018) and Shen *et al.* (2020) for additional computational details].

The qBIF procedure returns two aspects of the fitted SII model: (1) the BIF, with one spectral weight estimated for each frequency band in the filter bank and (2) a transfer function that links the SII value to proportion correct in keyword recognition. Following Du *et al.* (2019), the transfer function is defined as a logistic function,

$$P(\text{Correct})={\left[1+e^{-4\beta \left(\text{SII}-\theta \right)}\right]}^{-1},$$ (1)

where *θ* and *β* are two parameters that correspond to the SII value associated with 50% keyword recognition and how rapidly the recognition performance increases with increasing SII values, respectively.

In the current study, each qBIF run took approximately 15 min to complete and included 80 trials (i.e., 80 target sentences), broken into four blocks of 20 trials each. The total number of 80 trials was considered to provide sufficiently reliable estimates of the BIF according to previous studies that estimated the BIFs using the qBIF procedure and IEEE sentences (Shen *et al.*, 2020). The listener had the option to take short breaks between blocks or between qBIF runs. At the end of a qBIF run, estimates of the BIF, which included the spectral weights for the five octave-frequency bands, and the parameters of the transfer function [*θ* and *β*; see Eq. (1)] were obtained. The SII model implemented in the qBIF procedure was formulated so that the weights in the BIF always summed to unity. Therefore, each weight can be interpreted as the proportion of contribution from the corresponding frequency band.

For each listener, data collection was completed in two sessions. During the first session, four qBIF runs corresponding to the two speech materials and both the unmodulated and amplitude-modulated conditions were tested in random order. No target sentence was repeated within the session. At least one week after the first session, listeners returned for a second session during which the procedures in the first session were replicated.

For data analysis, the intra-subject variabilities in the estimated BIFs were examined between the repeated qBIF runs from the two test sessions. The effect of masker amplitude modulation on the shape of the BIF is evaluated using a repeated-measures analysis of variance (ANOVA). The estimated transfer functions under various stimulus conditions are used to confirm that FMB is observed in the current study and comparable to previous reports for similar test conditions. To facilitate these comparisons, the estimated transfer functions are used to derive the psychometric functions for speech recognition in speech-shaped noise using the same sentence materials. The predicted speech recognition threshold (SRT) is given by (30*θ* − 15) dB, and the psychometric-function slope is given by (100*β*/30)%/dB. Estimating these two parameters for sentence recognition in noise enables direct comparison to previous studies (e.g., MacPherson and Akeroyd, 2014).

## 3. Results

### 3.1 BIF

**Fig. 1.**
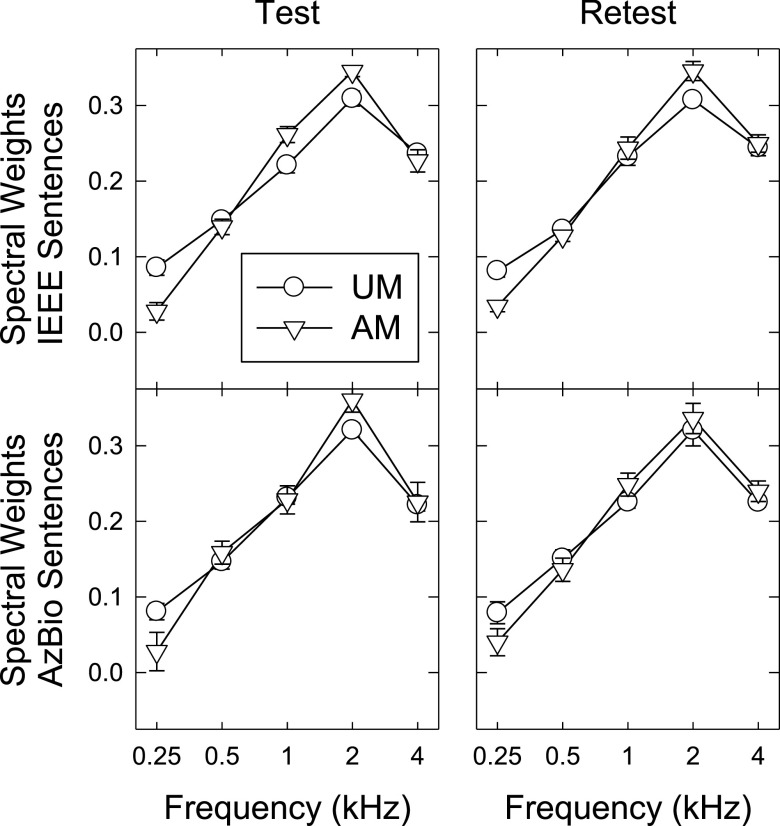
The mean BIFs estimated using co-located target and maskers. The top and bottom panels correspond to the IEEE and AzBio sentence materials, and the left and right panels show the two repeated estimates between the first (“Test”; left) and second (“Retest”; right) sessions. Within each panel, the BIFs estimated using unmodulated (UM) and amplitude-modulated (AM) maskers are shown using circles and triangles, respectively. Error bars indicate ± one standard error of the mean.

Figure 1 plots the estimated BIFs from the first (left) and second (right) sessions, averaged across the listeners. For both speech materials (arranged in the upper and lower panels) and both unmodulated (circles) and amplitude-modulated (triangles) maskers, the highest weights occurred for the 2-kHz band, and the weights decreased as the frequency moved further from 2 kHz. The lowest weights were observed at 250 Hz. This general shape resembled previously reported BIFs for sentence recognition using headphones (Shen *et al.*, 2020).

The two repeated estimates (left and right panels in Fig. 1) were close to each other. The root mean square deviations (RMSDs) between the estimated weights obtained from the first two sessions across frequency and conditions ranged from 0.025 to 0.084 (with a mean of 0.043 and a standard deviation of 0.016) for IEEE sentences and ranged from 0.042 to 0.113 (with a mean of 0.060 and a standard deviation of 0.021) for AzBio sentences. Therefore, there was a tendency that the test-retest reliability for the BIF estimates was better for the IEEE than the AzBio sentences [*t*(18) = 1.96, *p* = 0.065]. This may be related to the fact that the sentence length varied among individual AzBio sentences. Given the close resemblance of the two repeated qBIF runs, the averaged results across the test and retest were used in the subsequent analyses (see Table 1 for the average spectral weights).

**Table 1.** The estimated BIF (i.e., the spectral weights for the five octave-frequency bands) and the threshold and slope parameters of the transfer function (*θ* and *β*, respectively), averaged across the ten listeners. Results from various stimulus conditions are arranged in columns. The reported values are based on the average across two repeated qBIF runs during the first two sessions. In the table, each cell provides the mean result, followed by the standard error of the mean in parenthesis.

	IEEE		AzBio	
	UM^a^	AM^b^	UM	AM
Spectral weight				
0.25 kHz	0.083	0.031	0.080	0.034
	(0.007)	(0.006)	(0.011)	(0.019)
0.5 kHz	0.142	0.133	0.148	0.147
	(0.007)	(0.006)	(0.006)	(0.007)
1 kHz	0.227	0.252	0.228	0.238
	(0.006)	(0.009)	(0.007)	(0.008)
2 kHz	0.308	0.345	0.320	0.348
	(0.006)	(0.005)	(0.014)	(0.013)
4 kHz	0.240	0.238	0.223	0.233
	(0.007)	(0.011)	(0.004)	(0.017)
*θ*	0.268	0.219	0.240	0.190
	(0.010)	(0.012)	(0.007)	(0.010)
*β*	4.17	3.41	4.35	5.52
	(0.17)	(0.19)	(0.32)	(0.37)

^a^ Unmodulated (UM).

^b^ Amplitude-modulated (AM).

In Fig. 1, the effect of masker amplitude modulation on the shapes of the BIF is represented by the two different symbols within each panel. A repeated-measures ANOVA was conducted treating frequency (0.25, 0.5, 1, and 2 kHz), masker amplitude modulation, and speech material as the three independent variables and the average spectral weight across the test and retest as the dependent variable. Weights in the 4-kHz band were not included because the weights in a BIF always summed to unity (i.e., four degrees of freedom per BIF). There was a significant main effect of frequency [*F*(3, 27) = 552.56, *p* < 0.01, $\eta_{p}^{2}$ = 0.98], and there was a significant interaction between frequency and masker amplitude modulation [*F*(1.35, 12.17) = 9.57, *p* = 0.01, $\eta_{p}^{2}$ = 0.52, Greenhouse–Geisser corrected]. No other main effect or interaction reached statistical significance (*p* > 0.05). The significant interactions between frequency and amplitude modulation confirm the influences of masker envelope fluctuation on the shape of the BIF.

*Post hoc* paired comparisons found a significant increase in the spectral weights at 2 kHz [*t*(9) = 2.56, *p* = 0.03, Bonferroni corrected] and a significant decrease in the spectral weights at 0.25 kHz [*t*(9) = –3.97, *p* < 0.01, Bonferroni corrected] when the masker was amplitude-modulated. No significant difference associated with masker amplitude modulation was found at 0.5 or 1 kHz. Therefore, it seems that masker amplitude modulation enhanced the prominence of the 2-kHz peak in the BIF.

### 3.2 Psychometric functions: Observed FMB

The current study aimed to reveal listeners' spectral weighting under test conditions that are associated with FMB. Therefore, it is important to verify that FMB was observed in the current study and comparable to previously reported results. For this purpose, the psychometric functions for recognizing either the IEEE or AzBio sentences in speech-shaped noises were derived from the SII transfer function [Eq. (1)] estimated simultaneously with the BIFs using the qBIF procedure (the estimates of the transfer-function parameters *θ* and *β* are listed in Table 1).

**Fig. 2.**
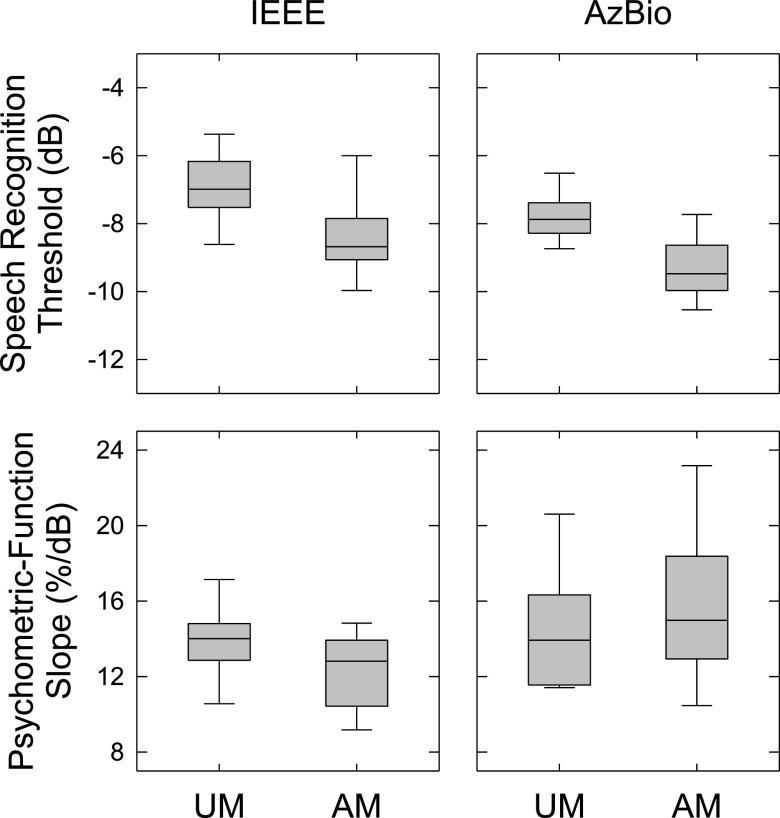
The SRTs (top panels) and psychometric-function slopes (bottom panels) for the IEEE (left) and AzBio (right) materials. For each panel, the various stimulus conditions are labeled along the *x* axis. UM and AM, the unmodulated and amplitude-modulated maskers, respectively. For each stimulus condition, estimates from the ten listeners are shown as a box plot indicating the 10th, 25th, 50th, 75th, and 90th percentiles.

Figure 2 shows the derived SRTs (top panels) and psychometric-function slopes (bottom panels). For the SRT, a repeated-measures ANOVA showed significant main effects of material [*F*(1, 9) = 22.13, *p* < 0.01, $\eta_{p}^{2}$ = 0.71] and masker amplitude modulation [*F*(1, 9) = 115.00, *p* < 0.01, $\eta_{p}^{2}$ = 0.93]. There was no significant interaction between material and masker amplitude modulation [*F*(1, 9) = 0.02, *p* = 0.90, $\eta_{p}^{2}$ = 0.00]. *Post hoc* paired comparisons showed that amplitude-modulating the masker caused an average decrease in SRT of 1.50 dB [*t*(9) = –10.72, *p* < 0.01, Bonferroni corrected], indicating an FMB. For the psychometric-function slope, a repeated-measures ANOVA showed a significant main effect of material [*F*(1, 9) = 7.86, *p* = 0.02, $\eta_{p}^{2}$ = 0.47]. There was no significant effect of masker amplitude modulation [*F*(1, 9) = 0.03, *p* = 0.87, $\eta_{p}^{2}$ = 0.00]; nor was there a significant interaction between material and masker amplitude modulation [*F*(1, 9) = 2.36, *p* = 0.16, $\eta_{p}^{2}$ = 0.21].

## 4. General discussion

The current study showed that the shape of the BIF for sentence recognition in noise maskers systematically depends on whether the masker was amplitude-modulated. When an 8-Hz amplitude modulation was introduced to the masker, an FMB was observed, and the shape of the estimated BIF changed. In particular, with an 8-Hz amplitude-modulated masker, the spectral weight at 2 kHz in the BIF increased, whereas the weight at 0.25 kHz decreased. Therefore, the spectral centroid of the BIF shifted slightly toward a higher frequency for the amplitude-modulated masker.

The observed effect of masker amplitude modulation on the shape of the BIF is not in line with the hypothesis that the additional use of the TFS cues across a wide frequency range may cause the spectral weights to be more evenly distributed across frequency for amplitude-modulated maskers compared to unmodulated maskers. This apparent lack of involvement of TFS cues could be related the range of the TMR tested during the qBIF procedure. The qBIF procedure adaptively adjusts the TMR in the various frequency bands so that the speech stimuli would be just intelligible to the listener. For this range of TMR, the TFS cues may not be sufficiently degraded to reveal its importance in glimpsing speech in amplitude-modulated maskers. Additionally, it is also likely that the TFS cues may not be as necessary for glimpsing the target in the current study because of the strong temporal grouping cue in the masker envelope provided by the 8-Hz amplitude modulation.

On the other hand, the shift of the spectral centroid of the BIF toward high frequencies in response to an amplitude-modulated masker is consistent with the hypothesis that the weights for individual phonemes may change under masker modulation. In particular, phonemes with high-frequency emphasis (near 2 kHz) may have been weighted as more important.

However, the observed shift in spectral weights may need to be interpreted with caution. This is because differences in the shape of the BIF between unmodulated and amplitude-modulated maskers may have arisen from the procedure to estimate the spectral weights. The spectral weights reported in the current study were estimated by fitting a correlational model to the experimental data, an established technique to reveal spectral weights (e.g., Doherty and Turner, 1996; Calandruccio and Doherty, 2007; Healy *et al.*, 2013; Bosen and Chatterjee, 2016; Shen and Kern, 2018). Such correlational analyses capture the average spectral weights over a number of test trials. If the listener's spectral weighting does not vary from trial to trial, then the correlational model would be able to capture the data well, leading to excellent goodness-of-fit. On the other hand, varying spectral weighting on a trial-by-trial basis would lead to a relatively poor goodness-of-fit and relatively small magnitudes for the correlation coefficients. In the qBIF procedure used in the current study, the spectral weights were obtained by normalizing the raw correlation coefficients from the logistic regression so that the weights summed to unity. This may cause the spectral weights to appear flat for a poorly fitted correlational model. Therefore, it is important to consider whether comparable goodness-of-fit was achieved for the unmodulated and amplitude-modulated masker conditions.

**Fig. 3.**
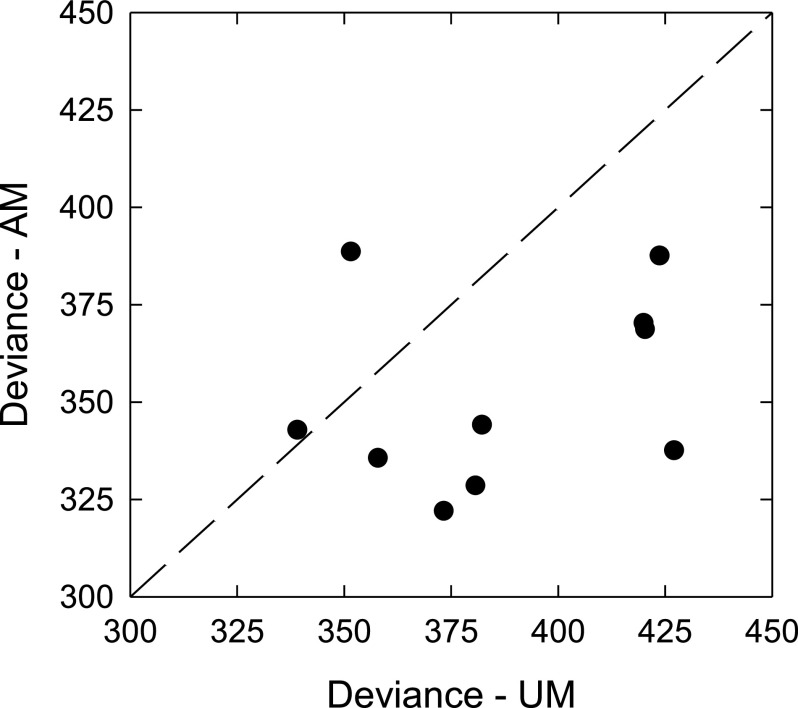
The deviances of the SII models in the unmodulated (UM) and amplitude-modulated (AM) masker conditions for the IEEE sentences.

To evaluate the goodness-of-fit of the logistic regression model for the unmodulated and amplitude-modulated conditions, for each test condition and each listener, the trial-by-trial data collected from the two test sessions using the IEEE sentences were pooled together and used to fit a single logistic regression model. The deviance of the model fit was derived. A higher deviance indicates a poorer goodness-of-fit. This analysis was not performed on data collected using the AzBio sentences because the number of data points used for model-fitting may vary across test conditions due to the varying sentence length of the AzBio corpus. Figure 3 plots the deviances calculated for the unmodulated (UM) and amplitude-modulated (AM) masker conditions. For the IEEE sentences, the deviance was larger for the unmodulated than the amplitude-modulated condition [*t*(9) = 3.22, *p* = 0.011], indicating that the goodness-of-fit for the logistic regression model was poorer for the unmodulated-masker condition. Therefore, it is possible that the listeners in the current study adopted more consistent spectral weights when the masker was amplitude-modulated compared to the steady-state masker. Consequently, whether the listeners shifted their spectral weights toward higher frequency regions cannot be confirmed with certainty at this time.

In a recent study, Buss and Bosen (2021) estimated the BIFs for disyllabic-word targets and two types of maskers: speech-shaped noise and two-talker speech. The BIF for the speech-shaped-noise masker exhibited a prominent peak at high frequencies (∼2.6 kHz), whereas the BIF for the two-talker-speech masker showed more distributed weights across a broader spectral range with frequencies near 1 kHz carrying the greatest importance. Therefore, it seems that the influence of an 8-Hz amplitude-modulated noise masker was different from that of a speech masker. This may be expected given that amplitude-modulating a noise masker typically improves speech recognition performance due to the introduction of glimpses of the target speech. On the other hand, a two-talker masker, even with a fluctuating envelope, may not necessarily facilitate speech recognition due to the potential involvement of informational masking (e.g., Freyman *et al.*, 2004; Leibold *et al.*, 2013; Schoof and Rosen, 2014; Buss *et al.*, 2021). Given the differences in the target speech material and the procedure to derive spectral weights between the two studies, the specific mechanisms that underlie the difference in the BIF between the amplitude-modulated noise and two-talker speech maskers cannot be identified at this time, which warrants future studies on this topic.

## 5. Summary

The current study demonstrated that the listeners' spectral weights while performing a speech recognition task using sentences depended on whether an 8-Hz amplitude modulation was applied to the competing background noise masker. Amplitude-modulating the masker increased the weight at 2 kHz and decreased the weight at 0.25 kHz. Findings from the current study warrant future studies that systematically investigate the dependences of spectral weighting during a speech recognition task on the temporal characteristics of the competing masker.
